# Cross-cultural adaptation and validation of the neurogenic bladder symptom score questionnaire for brazilian portuguese

**DOI:** 10.1590/S1677-5538.IBJU.2018.0335

**Published:** 2019-07-27

**Authors:** Lisley Keller Liidtke Cintra, José de Bessa, Victor Ikky Kawahara, Thereza Phitoe Abe Ferreira, Miguel Srougi, Linamara Rizzo Battistella, Daniel Rubio de Souza, Homero Bruschini, Cristiano Mendes Gomes

**Affiliations:** 1 Instituto de Medicina Física e Reabilitação, Faculdade de Medicina da Universidade de São Paulo - USP, São Paulo, SP, Brasil; 2 Divisão de Urologia da Faculdade de Medicina da Universidade de São Paulo - USP, São Paulo, SP, Brasil

**Keywords:** Urinary Incontinence, Urinary Bladder, Quality of Life

## Abstract

**Objective:**

To cross-culturally adapt and check for the reliability and validity of the neurogenic bladder symptom score questionnaire to Brazilian Portuguese, in patients with spinal cord injury and multiple sclerosis.

**Materials and Methods:**

The questionnaire was culturally adapted according to international guidelines. The Brazilian version was applied in patients diagnosed with neurogenic bladder due to spinal cord injury or multiple sclerosis, twice in a range of 7 to 14 days. Psychometric properties were tested such as content validity, construct validity, internal consistency, and test-retest reliability.

**Results:**

Sixty-eight patients participated in the study. Good internal consistency of the Portuguese version was observed, with Cronbach α of 0.81. The test-retest reliability was also high, with an Intraclass Correlation Coefficient of 0.86 [0.76 - 0.92] (p<0.0001). In the construct validity, the Pearson Correlation revealed a moderate correlation between the Portuguese version of the NBSS and the Qualiveen-SF questionnaire (r = 0.66 [0.40-0.82]; p <0.0001).

**Conclusions:**

The process of cross-cultural adaptation and validation of the NBSS questionnaire for the Brazilian Portuguese in patients with neurogenic lower urinary tract dysfunction was concluded.

## INTRODUCTION

Patients with neurogenic lower urinary tract dysfunction (NLUTD) constitute a population with several peculiarities. They often require special care in the management of their urinary problems, including the frequent need for intermittent bladder catheterization or for the use of an indwelling catheter. Thus, the use of unspecific questionnaires for the evaluation of lower urinary tract symptoms in this population is highly inaccurate (1).

Patient Reported Outcome Assessments (PROs) are effective tools for characterizing symptom burden and health-related quality of life (QoL) and they are playing an increasing role in clinical decision-making (2).

When selecting a PRO for use in a particular setting, it is of utmost importance to pick one that is relevant and applicable to that specific population and the desired evaluation or outcome. Once it has been decided which PRO to use, it is important to choose a questionnaire that has been scientifically developed and validated (1, 3).

Several instruments were developed and recommended for evaluating lower urinary tract symptoms invarying clinical conditions (4, 5) but, until recently, Qualiveen (6) was the only specific questionnaire to evaluate patients with NLUTD. It was developed primarily for evaluation of the QoL aspects related to urinary tract symptoms in patients with traumatic spinal cord injury and was later validated for patients with Multiple Sclerosis (7).

To meet the need for a questionnaire to assess lower urinary tract symptoms in patients with NLUTD, Welk et al. developed the Neurogenic Bladder Symptom Score (NBSS) (8). Considering the lack of any appropriate PRO to evaluate patients with NLUTD in Portuguese, we decided to translate, culturally adapt and validate the Neurogenic Bladder Symptom Score (NBSS) in Brazilian Portuguese.

## MATERIALS AND METHODS

### Cross-cultural adaptation

The cultural adaptation process was carried out according to international standards (9, 10), after authorization granted by the author.

Two versions of the NBSS were provided by two Portuguese-speaking individuals who were fluent in English, and an agreed-upon version in Portuguese was developed. The translated version was back-translated to English by a United States native, fluent in Portuguese, non-medical, blind to the purpose of the study. The authors reviewed the English back-translation to ensure that the original content had been maintained and to reveal inconsistencies with the Portuguese version. The results were submitted to a committee of bilingual members composed by two urologists specialized in voiding dysfunction, one urogynecologist and one physiatrist. The items of the questionnaire were analyzed by the judges as to their relevance and comprehensiveness of the aspects of the measured phenomenon. The committee also evaluated the semantic, idiomatic, and conceptual equivalence of each version item. The suggested modifications were performed only when the agreement percentage was approximately 90%. The author of the original study was informed of all stages of the study.

As a pilot/pre-test, the Brazilian version of the NBSS was applied to ten individuals (5 women and 5 men), aged 20 to 42 years, 5 with high school education and 5 with university/college education. In all cases, respondents were asked about the understanding of the questions and whether the alternatives were clear. After minor final adjustments made by the committee, the test version of the scale was concluded.

### Participants

Including criteria for the study were: minimum age of 18 years and diagnosis of NLUTD caused by spinal cord injury or multiple sclerosis. Exclusion criteria were: a history of urological surgery in the last 12 months, acute change in general health status during the study, patients with cognitive impairment, urinary tract infection in the last 30 days or recent change in the treatment of their NLUTD. These criteria were the same as those used by the researchers who developed the questionnaire (11).

Eligible patients were identified and invited to participate in the study from the daily schedule of medical consultations with the physiatrist or urologist. Sixty-eight patients of both genders with NLUTD were included in the study and all signed the informed consent form.

The study was approved by the Ethics Committee of the Hospital das Clínicas da Faculdade de Medicina da Universidade de São Paulo, # 1.177.224.

### Questionnaires

The NBSS is a comprehensive questionnaire for assessment of lower urinary tract symptoms and evaluation of consequences of NLUTD. It can be applied both in men and women with congenital or acquired NLUTD. The scale evaluates three domains that best represent the spectrum of neurogenic bladder dysfunction: 1- Incontinence (eight questions), 2- Storage and Voiding (seven questions) and 3- Consequences (seven questions). Two additional questions (first and last) are directed respectively to the method of bladder emptying and the impact of NLUTD on QoL and complete the 24 NBSS items. The NBSS has a possible score of 0 to 74, where the higher the score, the greater the severity of the symptoms. The authors validated each of the domains as an independent subscale, so that they can be used in combination or separately, according to clinical utility (11).

In most cases, the questionnaire was self-administered. Some patients, due to motor impairment for writing, visual acuity reduction or reading difficulties due to low educational level, asked the researchers for assistance in reading aloud or to mark the alternative of their choice. After answering the NBSS, participants were asked if they understood each of the questions and found suitable alternatives for them. In the occurrence of lack of comprehension of any question, or difficulty in identifying a suitable alternative for their case, the reason given by the patient was noted by the researcher in an appropriate file.

NBSS was reapplied after an interval of 7 to 14 days (12). At the second meeting, participants also answered the Qualiveen-SF QoL questionnaire (13).

The Qualiveen is the only questionnaire specific for patients with NLUTD, available in Portuguese (14). It consists of 08 questions, in the short form (SF) (13). It is divided into four domains: (1) Bother with limitations, (2) Fears, (3) Feelings, and (4) Frequency of limitations. The domains of Qualiveen-SF are composed of the combination of physical, emotional and social issues related to bladder dysfunction. The main focus of this questionnaire is on the psychosocial and functional impacts of the condition rather than the objective and quantifiable clinical symptoms (8).

In both encounters, participants answered a global bladder assessment question: “How much is the current functioning of your bladder a problem for you?” Using a Likert scale, participants chose a score of 0 to 5, with 0 or 1 indicated as a little problem, 2 or 3 moderate, and 4 or 5 very troublesome. Following the original validation study, a difference greater than or equal to two points between the first and second questionnaire responses was considered as a significant change in the individual's bladder function. These patients were not included in the test-retest reliability assessment.

Similarly to the original study, the following hypothesis was tested: Patients who answered the question of global evaluation of the bladder indicating a significant problem with bladder function should have an NBSS score significantly higher than those who indicated having moderate or no problem with the current functioning of the bladder.

### Psychometric Properties

According to recommendations for the process of cross-cultural adaptation (10), the following psychometric properties of the NBSS questionnaire were evaluated in our study:

1) Reliability, that refers to the degree to which the measurement is free from measurement error (15): In this study, we evaluated the measurement properties, internal consistency and test-retest reliability. Internal consistency is a measure that indicates how well items from the same subscale or domain correlate (12). It is evaluated by the Cronbach *a* coefficient, which must be greater than 0.7 to indicate good internal consistency (12). The test-retest reliability aims to show the reproducibility of the data comparing scores obtained on two different occasions without any therapeutic interventions between the two measurements. The results were assessed through the Intraclass Correlation Coefficient (ICC). A correlation above 0.7 is considered strong (12, 16). 2) Validity, that refers to the degree to which an outcome measure measures the construct it purports to measure (15): In this study, we evaluated the measurement properties construct and content validity. Construct validity refers to the instrument's ability to measure the built-in concept it intends to search for (17). It was assessed by Pearson's Correlation comparing the Qualiveen-SF questionnaire with the NBSS QoL question (Q24). The content validity is a subjective property tested by people with recognized knowledge in the subject, capable of judging whether the questions proposed in the scale are relevant and representative of all aspects of the phenomenon to be measured (17). It was determined by the committee of experts who reviewed and evaluated the NBSS.

The strength of correlations was evaluated according to standardized parameters, as follows (16). Downhill linear relationships have the same strength values, but negative: Exactly + 1. A perfect uphill (positive) linear relationship; +0.70. A strong uphill (positive) linear relationship;

+0.50. A moderate uphill (positive) relationship;

+0.30. A weak uphill (positive) linear relationship;

0. No linear relationship.

We also compared the total NBSS with the severity of the voiding symptoms measured by the global bladder assessment question. We tested for linear trend as a post-testing after one-way ANOVA.

We did not evaluate Responsiveness, which is a property that refers to the ability of an outcome measure to detect change over time in the construct to be measured (15).

### Statistical analysis

For the statistical analysis, we used MedCalc Statistical Software version 18.10.2 (MedCalc Software, Ostend, Belgium; http://www.medcalc.org; 2018).

## RESULTS

A total of 68 patients participated in the study, of which 57 (83.8%) were men, aged between 19 and 78 years, mean of 38.9. In 66 cases (97.1%), spinal cord injury was the cause of NLUTD. Table-1 shows the data of neurological disease time, lesion level, educational level and type of bladder management.

**Table 1:** Sociodemographic and clinical characteristics of 68 patients with neurogenic lower urinary tract disfunction.

Sociodemographic Characteristics			n (total = 68)
**Gender**			
	Male		83.8%
	Female		16.2%
**Age (years)**			
	Average (SD)	38.9	(± 14.7)
	Median (min-max)	35	(19-78)
**Education Level**			
	Illiterate	1	1.5%
	Elementary School complete or incomplete	16	23.5%
	High School complete or incomplete	38	55.9%
	College or more	12	17.6%
	Did not answer	1	1.5%
**Diagnosis**			
	Spinal Cord Injury	66	97.1%
	Multiple Sclerosis	1	1.5%
	Did not answer	1	1.5%
**Injury Time (months)**			
	Average (SD)	41	(± 42.6)
	Median (min-max)	27	(2-168)
**Injury Level**			
	Cervical	34	50.0%
	Thoracic	28	41.2%
	Low back	3	4.4%
	Sacral	1	1.5%
	Not identified or not applicable	2	2.9%
**ASIA Grade**			
	A	42	61.8%
	B, C, D or E	22	32.3%
	Not identified or not applicable	4	5.9%
**Bladder Management**			
	Indwelling catheter or urostomy bag	2	2.9%
	Condom catheter	0	–
	Intermittent catheter	59	86.8%
	Spontaneous voiding	7	10.3%

Of the 68 participants who completed the initial NBSS assessment, nine (13.2%) did not return for the retest. Thirteen patients (19.1%) who completed the retest questionnaire were excluded because they reported a significant change in bladder function between the first and second evaluation. Data from 46 patients who completed the test and retest phases were used for the test-retest reliability analysis. The percentage of blank responses was 2.0%.

For question 24, which evaluates the impact of NLUTD on QoL, the median score was 3 [2-5], indicating a predominance of patients dissatisfied with the QoL about their bladder condition. The median total score was 22 [15 to 30].

The internal consistency was tested by Cronbach *a* with a result of 0.81.

Forty-six patients participated in the test-retest reliability study which showed an ICC of 0.86 [0.76-0.92] (p < 0.0001) for the overall score. Table-2 presents the detailed results.

**Table 2:** Reproducibility and internal consistency of NBSS.

NBSS	ICC n = 46	CI 95%
Overall Score	0.86	0.76-0.92
Subdomains		
Incontinence	0.83	0.71-0.91
Storage and Voiding	0.81	0.75-0.90
Consequences	0.80	0.66-0.87

**CI** = Confidence Interval

Content validity was assessed through the subjective judgment of the expert committee (18), which confirmed that the scale covers the most relevant aspects related to NLUTD.

The construct validity was assessed in two ways. Pearson Correlation revealed a moderate correlation between NBSS with the Qualiveen-SF with a finding of 0.66 ([0.40-0.82]; p < 0.0001). We also evaluated this psychometric property comparing NBSS score with the value attributed to the overall bladder function. It should be noted that NBSS progressively and significantly increased as bladder function values. One-way ANOVA with trend analysis demonstrates that patients who reported having a more severe problem with their bladder function had higher NBSS scores (p= 0.026) (Table-3).

**Table 3:** Variation between the final score and the severity of the voiding symptoms (self-reported).

	NBSS final score	Trend to linearity
Response for global bladder function	Mean ± SD	
Little problem	18.51 ± 8.25	
Moderate	21.44 ± 8.94	P = 0.026
Very troublesome	26.32 ± 9.47	

**SD** = Standard Deviation

Of the 68 participants, 11 (16.2%) commented on the content of the questionnaire, including 13 comments about 11 questions. No comments were received from more than two (2.9%) participants.

Eleven observations (16.2%) were made about uncertainty as to the appropriate alternative for their case of some of the questions, and two other comments (2.9%) were about difficulty of understanding a term or suggestion for improvement.

For the analysis of the observations about uncertainty as to the appropriate alternative for their case, we tried to evaluate the impact of the educational level. Participants were divided into two groups based on having complete or incomplete high school level education, and it was not possible to establish an association between educational level and the emergence of misunderstandings (p = 0.47).

The observations about incomprehension were low and scattered among the questions, which attests that the instrument is adequate and does not need more changes.

## DISCUSSION

Evaluation questionnaires are developed largely in English (19). To use assessment tools created in a foreign language, a systematic and standardized process of cultural adaptation and validation is necessary to guarantee the success of the version, considering linguistic and cultural differences (10). This process allows the achievement of benchmarks and comparisons with international data, also, to opening doors for multicenter trials involving different countries (20).

The NBSS was developed to meet clinical and scientific needs for an instrument to assess symptoms associated with NLUTD and its consequences (8). The NBSS items were developed through interviews with patients (with a diagnosis of spinal cord injury, multiple sclerosis, and spina bifida), expert opinion and a literature review (1). It has been shown to be responsive to positive clinical changes and may be used to monitor patient improvement over time or after intervention (21). It is also available in Italian (22).

The process of cultural adaptation of the NBSS followed an internationally recognized methodology, proposed by Beaton et al. (10) and Guillemin et al. (9). This method has been successfully applied in Brazil in other similar works (19, 23, 24).

Most of the patients answered the questionnaire by themselves, without the help of the researchers. In this case, some items are expected to be left blank (25). Notwithstanding, the percentage of blank responses in this study was very low (2.0%). The comprehension analysis demonstrated that the NBSS is a self-applicable questionnaire, simple and easy to answer.

The present study demonstrated the following psychometric properties of the adapted NBSS version: content validity, internal consistency, test-retest reliability, and construct validity.

In the internal consistency analysis, the Cronbach α of 0.81 indicated a good level of internal consistency, similar to the result obtained in the original NBSS development work, which was 0.89 (11). In the evaluation of reliability, a strong correlation (0.86) was observed, also similar to that obtained in the original study (0.91) (11). When evaluated separately, each of the subscales also showed strong test-retest reliability.

The construct validity was performed through the QoL questionnaire Qualiveen-SF. Its correlation with the NBSS QoL question (Q24) was investigated, in the same way as the original study. In our study, the correlation with the Qualiveen-SF was moderate (0.66), very similar to the result found in the original English version (0.67) (11). This moderate correlation is possibly due to the variability imposed by measuring quite diverse constructs in their essence (18). While Qualiveen-SF was developed for QoL assessment, the NBSS focuses on assessing symptoms and their achievements, with only one question for QoL assessment.

The construct validity was also demonstrated in our analysis comparing the total NBSS score and the question of overall bladder evaluation. The same was tested and proven in the original study. Additionally, it is possible to notice a trend of linearity: the higher the score, the worse the patient's perception of their bladder function.

A potential limitation of our study is the sample size. Although it is crucial for the planning of studies, there is no consensus on the ideal sample for cross-cultural adaptation and validation studies. van Nispen et al. (26) suggested that the existing recommendations (10, 20) do not properly define what an adequate sample size is and proposed that since this issue is highly dependent on the construct to be measured, users should make this decision for their application. Terwe et al. (27) stated that no criteria have been defined for the required sample size of studies assessing measurement. Likewise, they consider a sample size of at least 50 patients adequate to determine the minimal important changes to evaluate interpretability. Anthoine et al. (28) in a review article showed that the sample size determination for psychometric validation studies is rarely ever justified a priori and that 86% of the validation studies did not provide robust justification for their sample size. Finally, in a recent study by Danielsen et al. (29), the authors extensively reviewed the methodological process of studies focused on translation and validation of questionnaires from English to an European language. They found that most researchers did not use a translation guide and that the only consensus was centered on the use of forward/backwards-translations and use of Cronbach's alpha when testing the internal validity. They also criticized the available recommendations and guidelines stating that they do not seem to cover both the translation as well as testing of the translation. This study further emphasizes the lack of clear scientifically sound recommendations on this topic.

Although patients with NLUTD are frequent in rehabilitation centers, it is not easy to enroll a large sample population for studies. Their associated mobility and transportation restrictions (most are wheelchair bound) make it difficult for them to participate in studies that require more than one meeting, in a brief period of time (14).

We believe that despite the relatively small sample in our study, we were able to properly perform the cross-cultural adaptation and validation of the NBSS questionnaire for the Brazilian Portuguese (Appendix). Our sample meets the minimum criteria proposed by Terwee et al. (27) and is equivalent to several other validation studies (14, 19). Although the recommendation by Beaton (10) is for a larger population, it has become acceptable in many studies to include smaller samples, especially when the studied population is not very large and easy to enroll.

In our sample, most patients (97.1%) had a diagnosis of spinal cord injury, characteristic to the profile of the institutions where the data were collected. There was a predominance of men (83.8%), reflecting the prevalence of this gender in the spinal cord-injured population (30), followed by spinal cord injury (35%) and congenital NLUTD (6%). Despite the difference between the samples from the two studies, the results were very similar.

## CONCLUSIONS

The results of this study demonstrated that the Brazilian version of the Neurogenic Bladder Symptom Score has good internal consistency and reliability, with clear and relevant content that is appropriate to measure the correct construct.

We considered that the process of cultural adaptation and validation of the NBSS Portuguese questionnaire for patients with neurogenic lower urinary tract dysfunction was performed. The Brazilian version of the NBSS is suitable for clinical and research use.

## ABBREVIATIONS

CI: Confidence Interval
ICC: Intraclass Correlation Coefficient
NBSS: Neurogenic Bladder Symptom Score
NLUTD: Neurogenic lower urinary tract dysfunction
PRO: Patient Reported Outcome Assessment
QoL: Quality of life
SD: Standard Deviation
SEM: Standard Error of Measurement
SF: Short Form

## Appendix Portuguese version of the NBSS and the Qualiveen-SF questionnaire.

Data: ____/____/____

ID do Paciente: ________________

### AVALIAÇÃO DE SINTOMAS DA BEXIGA NEUROGÊNICA

- Este questionário é sobre problemas urinários que você pode ter.
- Por favor, responda a todas as questões escolhendo apenas uma alternativa como resposta.
- O tempo para responder este questionário é de aproximadamente 5 a 10 minutos.
- Ao responder, considere o funcionamento habitual da sua bexiga. Não inclua alterações temporárias/passageiras da bexiga, tais como infecção urinária recente.
- Todas as questões podem ser respondidas tanto por pacientes que urinam espontaneamente quanto pelos que usam sondas, cateteres ou bolsas coletoras ou que já foram submetidos à cirurgia de bexiga.
